# Clinical Outcomes and Learning Curve of Endoscopic Ultrasound‐Guided Hepaticogastrostomy During the Implementation Phase in Inexperienced Centers: A Multicenter Retrospective Study

**DOI:** 10.1002/deo2.70291

**Published:** 2026-01-31

**Authors:** Junichi Kaneko, Tatsunori Satoh, Yosuke Kobayashi, Azumi Suzuki, Shinya Kawaguchi

**Affiliations:** ^1^ Department of Gastroenterology Iwata City Hospital Shizuoka Japan; ^2^ Department of Gastroenterology Shizuoka General Hospital Shizuoka Japan; ^3^ Department of Gastroenterology Seirei Hamamatsu General Hospital Shizuoka Japan; ^4^ Department of Gastroenterology Hamamatsu Medical Center Shizuoka Japan

**Keywords:** biliary drainage, EUS‐HGS, EUS‐BD, hepaticogastrostomy, inexperienced centers

## Abstract

**Objectives:**

Endoscopic ultrasound‐guided hepaticogastrostomy (EUS‐HGS) is a technically demanding procedure performed predominantly at centers with high expertise. Its feasibility and learning curves in inexperienced centers remain unclear. This study aimed to evaluate the initial clinical outcomes and learning curves of EUS‐HGS implemented in inexperienced centers.

**Methods:**

Between September 2018 and December 2020, four tertiary care centers specializing in pancreaticobiliary disease implemented EUS‐HGS. The first 20 patients who underwent EUS‐HGS at each institution were retrospectively enrolled. The primary outcomes were technical success, procedure time, and serious (moderate‐to‐severe) adverse events (AEs). A learning curve analysis was conducted using chronological quartiles. Logistic regression was used to identify the predictors of technical failure and serious AEs.

**Results:**

The overall technical success rate was 94% (75/80); serious AEs occurred in 11% (9/80). Procedure time significantly decreased across quartiles, whereas no significant trend was observed for technical success or serious AE rates. The univariate analysis did not identify significant predictors of technical failure, whereas the multivariate analysis identified ascites as an independent predictor of serious AEs.

**Conclusions:**

EUS‐HGS can be feasibly implemented with caution in inexperienced centers, particularly when performed by endoscopists with adequate pancreatobiliary expertise. Additionally, during the implementation phase, procedure time decreased with increasing institutional experience.

## 1 Introduction

Endoscopic ultrasound‐guided biliary drainage (EUS‐BD) is a promising alternative to percutaneous transhepatic biliary drainage in patients with malignant biliary obstruction following failed endoscopic retrograde cholangiopancreatography (ERCP) [1, 2]. EUS‐BD includes endoscopic transmural stent placement techniques, such as EUS‐guided choledochoduodenostomy (EUS‐CDS) and EUS‐guided hepaticogastrostomy (EUS‐HGS) [3, 4]. EUS‐CDS is not feasible in patients with duodenal obstruction or surgically altered upper gastrointestinal anatomy; hence, EUS‐HGS serves as a more versatile alternative in clinical practice [5]. EUS‐HGS is technically demanding and predominantly performed in expert centers. In inexperienced centers, the clinical outcomes have been unsatisfactory; a previous multicenter retrospective study reported a technical success rate of only 65% with an adverse event (AE) rate of 29% [6]. Since its introduction over a decade ago, EUS‐HGS has evolved considerably, with improved techniques and dedicated devices, contributing to enhanced safety [7, 8, 9, 10]. Recent systematic reviews have reported technical success rates of approximately 94%, clinical success rates of 89%, and AE rates of 24%, with 20% classified as moderate or severe [11]. However, these outcomes are largely derived from studies conducted at expert centers and may be subject to publication bias, limiting their generalizability to inexperienced centers. A recent multicenter retrospective study conducted across 22 inexperienced centers in Japan demonstrated favorable outcomes for EUS‐BD, with a technical success rate of 91.4% and an AE rate of 10.2% [12]. However, no studies have examined the clinical outcomes of EUS‐HGS during the implementation phase, including the learning curves. To address this gap, we conducted a multicenter retrospective study to evaluate the outcomes of the first 20 EUS‐HGS cases, assess the learning curve, and verify the feasibility of EUS‐HGS in inexperienced centers.

## 2 Methods

### 2.1 Study Design

Four institutions in Shizuoka Prefecture, Japan, namely Shizuoka General Hospital, Seirei Hamamatsu General Hospital, Hamamatsu Medical Center, and Iwata City Hospital, participated in this study. These institutions, all tertiary care centers specializing in pancreaticobiliary disease, introduced EUS‐HGS between September 2018 and December 2020. We retrospectively reviewed the electronic medical records of the first 20 patients who underwent EUS‐HGS at each institution from its introduction through July 2024, totaling 80 patients. The need for informed consent was waived using the opt‐out method. This study was approved by the Institutional Review Board of Iwata City Hospital (approval number of the Institutional Review Board: 2024–934) and was conducted in accordance with the principles of the Declaration of Helsinki.

### 2.2 EUS‐HGS Procedure

Each EUS‐HGS operator had extensive experience, including over 1000 ERCP procedures and substantial EUS experience (≥436 EUS examinations and ≥93 EUS‐guided fine‐needle aspiration procedures), which are factors associated with improved EUS‐BD outcomes [13]. However, all operators had limited previous experience with EUS‐HGS (<5 cases) but gained a deeper understanding of EUS‐HGS through clinical practice or on‐site observation at expert centers. In three institutions, a single operator mainly performed the procedures, whereas in one institution, they were mainly performed by two operators.

All procedures were performed using a curved linear array echoendoscope (GF‐UCT260; Olympus Medical Systems Corp., Tokyo, Japan) under conscious sedation. The intrahepatic bile duct (B2 or B3) was visualized and punctured, followed by cholangiography, guidewire advancement, contrast catheter insertion (with bile aspiration and additional cholangiography), tract dilation, and stent deployment. Procedural details, including the use of additional antegrade stenting and the selection of stent type, were determined at the discretion of the attending doctor based on the patient's clinical condition.

### 2.3 Outcomes and Definitions

The primary outcomes of this study were technical success, procedure time, and incidence of serious AEs, which were evaluated to assess the learning curve. In addition, predictors of technical failure, prolonged procedure time (≥ third quartile), and serious AEs were analyzed.

Technical success was defined as the successful deployment of the transmural stent. Procedure time was defined as the interval between the insertion of the EUS scope and the completion of HGS stent deployment. AEs were defined as any complications within 14 days of EUS‐HGS, including bile leakage, biliary peritonitis, perforation, bleeding, cholecystitis, cholangitis, liver abscess, pancreatitis, pneumonia, and stent migration. AE severity was graded according to the American Society for Gastrointestinal Endoscopy guidelines [14]. Moderate or severe AEs were considered serious. Clinical success was defined as, within 7 days after the procedure, a ≥50% reduction or normalization of total bilirubin for patients with jaundice, a sufficient reduction or normalization of target liver enzymes for patients with elevated levels of other liver enzymes, or resolution of cholangitis (cessation of antibiotics or a ≥50% reduction or normalization of blood inflammatory markers) for patients with cholangitis [15].

Ascites was defined as the presence of fluid on preprocedural imaging in areas such as the peri‐hepatic space or the hepato‐gastric recess, excluding minimal fluid confined to the pelvic cavity owing to its limited clinical significance. The distance to the hepatic parenchyma was measured from the puncture site of the intrahepatic bile duct to the periphery of the hepatic parenchyma using EUS imaging.

### 2.4 Statistical Analysis

Continuous variables are expressed as medians and interquartile ranges (IQRs), and categorical variables as counts and percentages. Categorical variables were compared using the chi‐squared test. To evaluate the learning curve, 20 patients at each institution were allocated into four chronological quartiles (Cases 1–5, 6–10, 11–15, and 16–20). Trends in technical success and serious AE rates across these quartiles were assessed using the Cochran–Armitage trend test. Procedure time trends across chronological quartiles were assessed using the Jonckheere–Terpstra test, which detects ordered differences in medians across multiple groups. For the sensitivity analysis, procedural outcomes were evaluated by dividing the cases into five chronological quintiles (Cases 1–4, 5–8, 9–12, 13–16, and 17–20).

The predictors of technical failure, prolonged procedure time (≥ third quartile), and serious AEs were analyzed using logistic regression analysis. Continuous variables were dichotomized using optimal cutoff values determined using receiver operating characteristic curve analysis. Variables that could not be analyzed owing to separation or insufficient variability (e.g., all cases falling into a single outcome category) were excluded from the regression analysis. Where appropriate, variables with *p* < 0.10 in univariate analysis were included in a multivariate logistic regression model to identify independent predictors. A *p*‐value of <0.05 was considered statistically significant. All statistical analyses were performed using EZR (Saitama Medical Center, Jichi Medical University), a graphical user interface for R (R Foundation for Statistical Computing, Vienna, Austria) [16].

## 3 Results

### 3.1 Patient and Procedure Characteristics

The first 20 patients who underwent EUS‐HGS at each participating institution were enrolled, resulting in 80 patients in the final analysis. Patient and procedural characteristics are summarized in Table 1. The most common primary disease was pancreatic cancer (43 patients; 54%). Ascites was observed in eight patients (10%); however, no cases demonstrated ascites along the puncture tract. Transmural metallic stents were placed in 39 patients (49%), whereas transmural plastic stents were placed in 36 (45%). Antegrade stenting was performed in 24 (30%) patients.

**TABLE 1:** Clinical and procedure characteristics (*n* = 80).

Age, year, median (IQR)	77 (71–82)
Sex, male, *n* (%)	43 (54)
PS, 0/1/2/3, *n* (%)	16 (20)/43 (54)/16 (20)/5 (6)
Primary disease, *n* (%)	
Pancreatic cancer	43 (54)
Biliary cancer	21 (26)
Other neoplasms	16 (20)
Site of biliary obstruction, *n* (%)	
Distal part	62 (78)
Hilar part	18 (23)
Indications for EUS‐HGS, *n* (%)	
Duodenal invasion/obstruction	28 (35)
Difficult biliary cannulation during ERCP	28 (35)
Postsurgical altered anatomy	15 (19)
Failure to achieve adequate biliary drainage by ERCP	9 (11)
Ascites, *n* (%)	8 (10)
Ascites along the puncture tract	0 (0)
Preprocedural cholangitis, *n* (%)	31 (39)
Mild/moderate/severe	15/14/2
Puncture site, *n* (%)	
B3/ B2	64 (80)/16 (20)
Bile duct diameter, median (IQR), mm,	6 (5–8)
Distance to the hepatic parenchyma, median (IQR), mm	22 (18–30)
Needles, *n* (%)	
19‐gauge	72 (90)
22‐gauge	8 (10)
Dedicated Equipment for tract dilation, *n* (%)	78 (98)
Balloon dilator	34 (43)
Tapered mechanical dilator	25 (31)
Cautery‐tipped dilator	9 (11)
Only ERCP catheter	10 (13)
Antegrade stenting, *n* (%)	24 (30)
Transmural stent placement, *n* (%)	75 (94)
Metal stent	39 (49)
Partially covered stent	16 (20)
6 mm × 12 cm/8 mm × 10 cm/8 mm × 12 cm	3/1/12
Partially covered stent with anti‐migration properties	18 (23)
8 mm × 10 cm/8 mm × 12 cm	9/9
Fully covered stent with thin delivery system	5 (6)
6 mm × 10 cm/6 mm × 12 cm/8 mm × 12 cm	1/1/3
Plastic stent, 7fr	36 (45)

Abbreviations: ERCP, endoscopic retrograde cholangiopancreatography; IQR, interquartile range; PS, performance status.

### 3.2 Overall Clinical Outcomes

**FIGURE 1:**
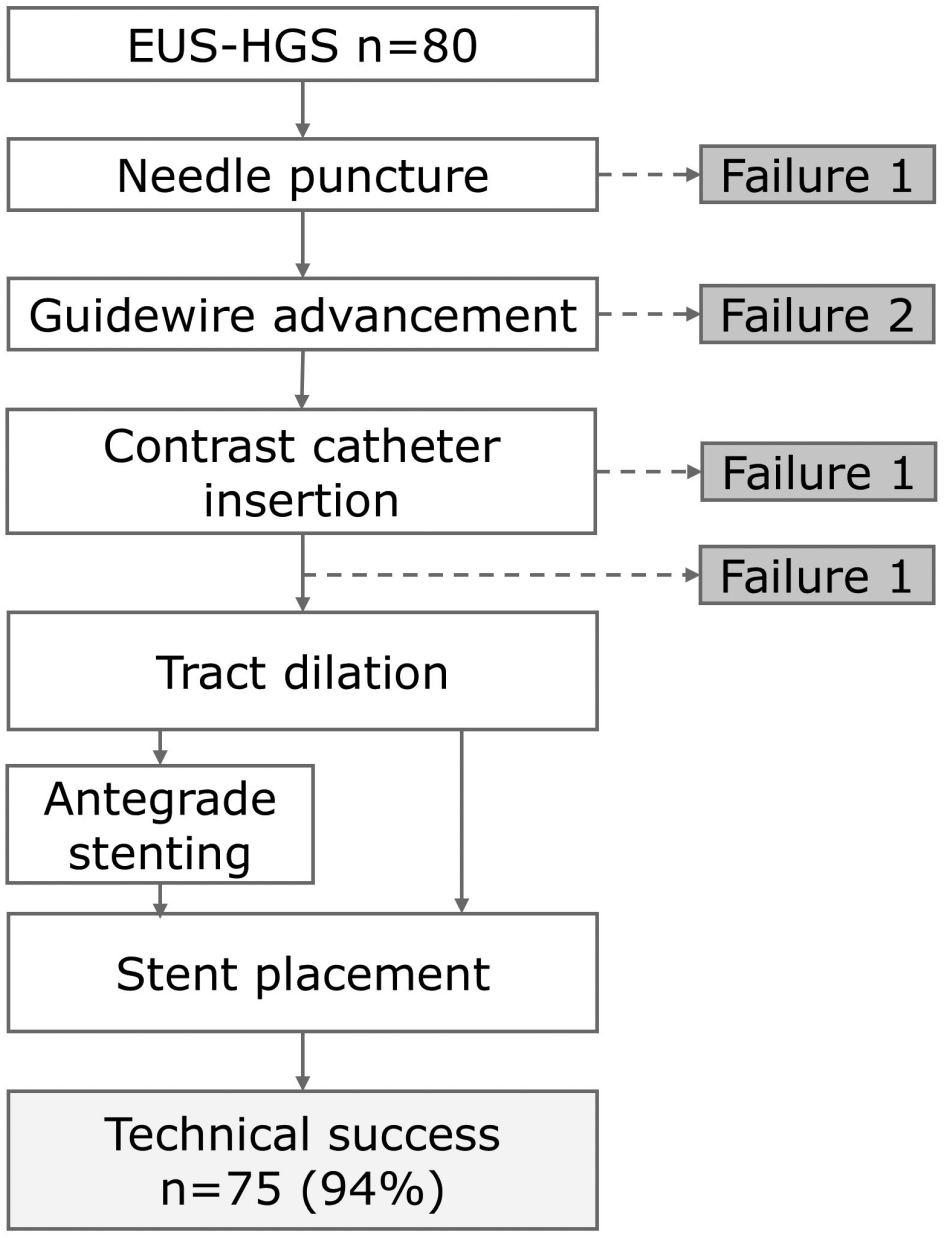
Flow diagram for the technical process of endoscopic ultrasound‐guided hepaticogastrostomy (EUS‐HGS).

Overall clinical outcomes are shown in Table 2. The technical success rate was 94%. Among the five cases with technical failure, the failure points occurred at different procedural steps: one failed at the puncture step, two failed during guidewire advancement, one case failed during contrast catheter insertion, and one procedure was aborted due to sudden respiratory arrest despite successful completion of the initial procedural steps before tract dilation (Figure 1). The median procedure time was 34.5 min (IQR, 25.8–45.0). Serious AEs occurred in nine patients (11%): bleeding in three, biliary infection (cholangitis and cholecystitis) in three, peritonitis in two, and pneumonia in one. Serious AEs included nine cases of moderate severity. The onset was immediate/intra‐procedural in four patients and delayed (≥24 h) in five patients. These required additional interventions (additional endoscopic intervention in three patients, percutaneous intervention in two, only blood transfusion in one, additional endoscopic intervention plus blood transfusion in one, and conservative treatment in two). No unplanned intravascular or surgical intervention was required. Details of the cases with technical failure and serious AEs are provided in Table S1. The clinical success rate was 85% (68/80).

**TABLE 2:** Overall outcome of endoscopic ultrasound‐guided hepaticogastrostomy (EUS‐HGS) (n = 80).

Technical success, *n* (%)	75 (94)
Procedure time, minutes, median (IQR)	34.5 (25.8–45.0)
Serious AEs [moderate]/ overall AEs, *n* (%)	9 (11) [9]/17 (21)
Details of serious AEs	
Bleeding/Biliary infection/Peritonitis/Pneumonia, n	3/3/2/1
Clinical success, *n* (%)	68 (85)

Abbreviations: AE, adverse event; EUS‐HGS, endoscopic ultrasound‐guided hepaticogastrostomy; IQR, interquartile range.

### 3.3 Learning Curve Analysis

**FIGURE 2:**
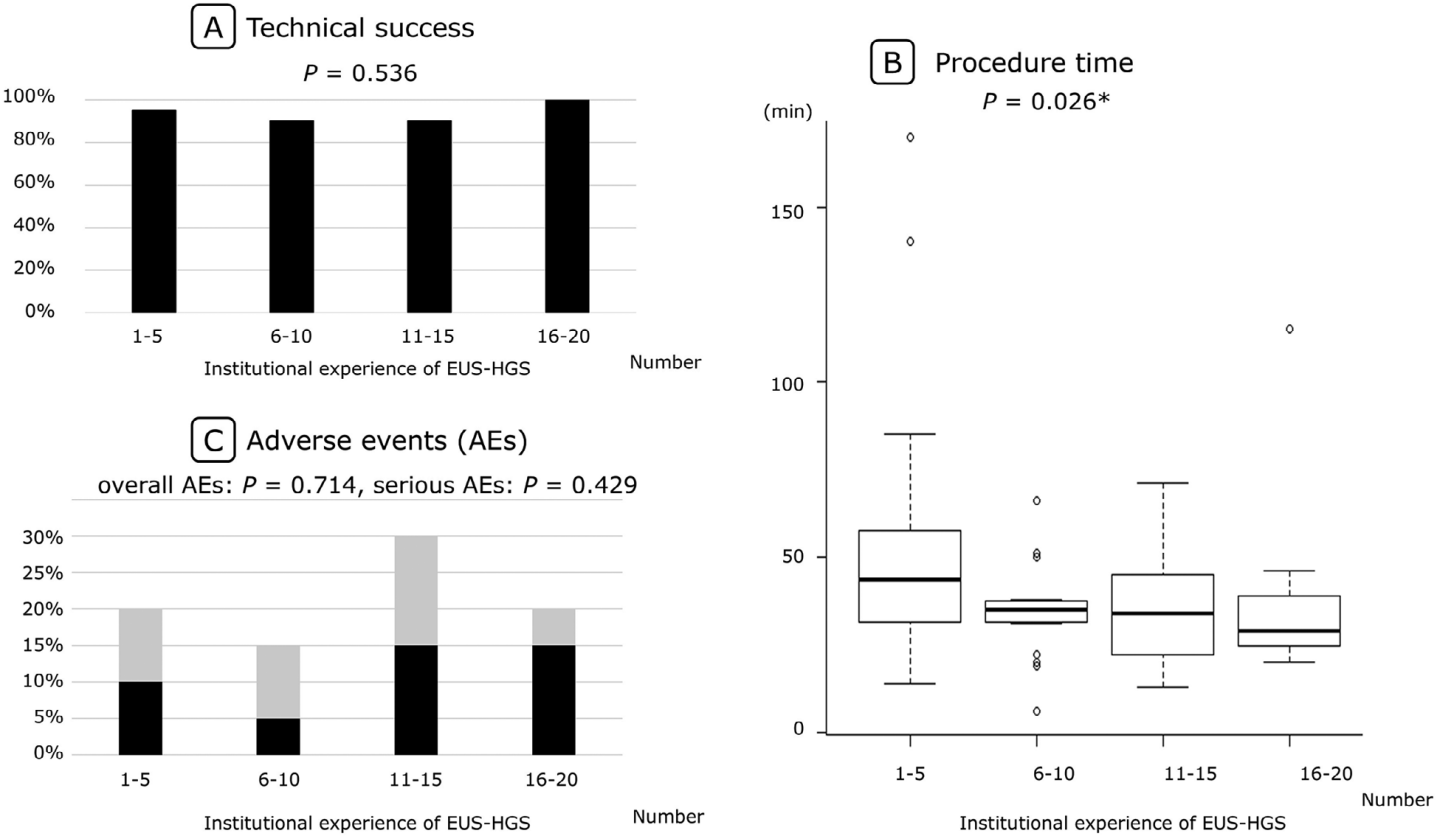
Institutional learning curve of endoscopic ultrasound‐guided hepaticogastrostomy (EUS‐HGS) across chronological quartiles: graph showing the relationship between technical success (A), procedure time (B), or adverse events (C) and institutional experience using four chronological quartiles (cases 1–5, 6–10, 11–15, and 16–20). In (C), the overall events are indicated in black, whereas serious adverse events are indicated in gray.

Twenty cases from each institution were allocated into four chronological quartiles (cases 1–5, 6–10, 11–15, and 16–20) to assess the learning curve. Technical success rates were 95%, 90%, 90%, and 100% across the four quartiles, showing no significant trend (*p* = 0.536) (Table 3 and Figure 2A). The median procedure time decreased from 44 min in the first quartile to 29 min in the last quartile (*p* = 0.026) (Table 3 and Figure 2B). In addition, the serious AE rates were 10%, 5%, 15%, and 15% across the four quartiles, showing no significant trend (*p* = 0.429) (Table 3 and Figure 2C). The overall AE rates were 20%, 15%, 30%, and 20% across the four quartiles, showing no significant trend (*p* = 0.714) (Table 3 and Figure 2C).

**TABLE 3:** Learning curve of endoscopic ultrasound‐guided hepaticogastrostomy (EUS‐HGS) using four chronological quartiles.

No. of institutional case	1–5 (*n* = 20)	6–10 (*n* = 20)	11–15 (*n* = 20)	16–20 (*n* = 20)	*p*‐value
Technical success, *n* (%)	19 (95)	18 (90)	18 (90)	20 (100)	0.536
Procedure time, minutes, median (IQR)	44 (32–57)	35 (32–37)	34 (22–45)	29 (25–39)	0.026*
Serious AEs	2 (10)	1 (5)	3 (15)	3 (15)	0.429
Overall AEs, *n* (%)	4 (20)	3 (15)	6 (30)	4 (20)	0.714

Abbreviations: AE, adverse event; EUS‐HGS, endoscopic ultrasound‐guided hepaticogastrostomy; IQR, interquartile range.

* *p* < 0.05.

The sensitivity analysis using five chronological quintiles confirmed a reduction in procedure time with increasing institutional experience, while no clear association was observed with technical success or serious AE rates (Figure S1).

### 3.4 Predictors of Technical Failure, Prolonged Procedure Time, and Serious AEs

Univariate analysis was performed to identify the potential predictors of technical failure. Owing to the limited number of events (n = 5), a multivariate analysis was not conducted. None of the variables were statistically significant (Table 4).

**TABLE 4:** Factors affecting technical failure of endoscopic ultrasound‐guided hepaticogastrostomy (EUS‐HGS).

	Technical success	Technical failure	Univariate analysis	
Variables	(*n* = 75)	(*n* = 5)	OR (95% CI)	*p‐*value
Age ≤ 79 years	46	4	2.52 (0.27–23.70)	0.418
Male sex	40	3	1.31 (0.21–8.31)	0.773
PS ≥ 2	20	1	0.688 (0.07–6.52)	0.744
Pancreatic cancer	39	4	3.69 (0.39–34.60)	0.253
Preprocedural cholangitis	30	1	0.38 (0.04–3.52)	0.391
Ascites	7	1	2.43 (0.24–24.80)	0.455
Bile duct diameter ≤ 6 mm	38	4	3.89 (0.42–36.50)	0.234
Institutional experience of EUS‐HGS ≤ 10 cases	37	3	1.54 (0.24–9.75)	0.646
B2 puncture	15	1	1.00 (0.10–9.61)	1.000
Distance to the hepatic parenchyma ≤ 25 mm	49	3	0.80 (0.13–5.07)	0.809

Abbreviations: CI, confidence interval; EUS‐HGS, endoscopic ultrasound‐guided hepaticogastrostomy; OR, odds ratio; PS, performance status.

Univariate analysis also revealed that institutional experience of ≤5 cases was the only factor significantly associated with prolonged procedure time (≥45 min). Because no other variables reached statistical significance, multivariate analysis was not performed (Table 5).

**TABLE 5:** Factors affecting prolonged procedure time of endoscopic ultrasound‐guided hepaticogastrostomy (EUS‐HGS).

	Procedure time <45 min	Procedure time ≥45 min	Univariate analysis	
Variables	(*n* = 59)	(*n* = 21)	OR (95% CI)	*p‐*value
Hilar biliary obstruction	13	5	1.11 (0.34–3.59)	0.867
Preprocedural cholangitis	24	7	0.729 (0.26–2.07)	0.554
Needles, 22 gauge	5	3	1.80 (0.39–8.29)	0.451
Antegrade stenting	18	6	0.91 (0.30–2.73)	0.868
Metal stents for HGS	29	10	1.21 (0.42–3.50)	0.729
Institutional experience of EUS‐HGS ≤ 5 cases	12	9	3.27 (1.11–9.68)	0.032*
Operator's experience of EUS‐HGS ≤ 5 cases	17	4	1.31 (0.36–4.80)	0.686

Abbreviations: CI, confidence interval; EUS‐HGS, endoscopic ultrasound‐guided hepaticogastrostomy; OR, odds ratio.

* *p* < 0.05.

In addition, univariate analysis revealed an association between ascites (odds ratio [OR], 6.60; 95% confidence interval [CI], 1.26–34.60, *p* = 0.026) and serious AEs, and multivariate analysis identified ascites as an independent predictor of serious AE (OR, 6.32; 95% CI, 1.13–35.60, *p* = 0.036) (Table 6).

**TABLE 6:** Factors affecting serious adverse events (AEs) of endoscopic ultrasound‐guided hepaticogastrostomy (EUS‐HGS).

	No serious AEs	Serious AEs	Univariate analysis		Multivariate analysis	
Variables	(*n* = 71)	(*n* = 9)	OR (95% CI)	*p‐*value	OR (95% CI)	*p‐*value
Age ≤ 79 years	42	8	5.52 (0.66–46.60)	0.116		
Male sex	38	5	1.09 (0.27–4.38)	0.908		
PS ≥ 2	20	1	0.32 (0.04–2.71)	0.295		
Pancreatic cancer	38	5	1.09 (0.27–4.38)	0.908		
Hilar biliary obstruction	16	2	0.982 (0.19–5.20)	0.983		
Preprocedural cholangitis	27	4	1.30 (0.32–5.28)	0.710		
Ascites	5	3	6.60 (1.26–34.60)	0.026*	6.34 (1.13–35.60)	0.036*
Bile duct diameter ≤ 6 mm	36	6	1.94 (0.45–8.39)	0.373		
Institutional experience of EUS‐HGS ≤ 10 cases	37	3	0.46 (0.11–2.00)	0.297		
B2 puncture	13	3	2.23 (0.49–10.10)	0.298		
Distance to the hepatic parenchyma ≤ 25 mm	47	5	0.64 (0.16–2.60)	0.531		
Metal stents for HGS	36	3	0.67 (0.14–3.21)	0.613		
Procedure time ≥ 42 min	20	5	3.19 (0.78–13.10)	0.108		
Technical failure	3	2	6.48 (0.92–45.60)	0.061	6.10 (0.77–48.70)	0.088

Abbreviations: AE, adverse event; CI, confidence interval; EUS‐HGS, endoscopic ultrasound‐guided hepaticogastrostomy; OR, odds ratio; PS, performance status.

* *p* < 0.05.

## 4 Discussion

This study assessed the initial outcomes of EUS‐HGS performed in inexperienced centers, including a learning curve analysis. The overall outcome of EUS‐HGS showed a technical success rate of 94% and a serious AE rate of 11% (overall AE rate of 21%). Procedure time decreased with increasing institutional experience in EUS‐HGS; however, no correlation was found between institutional experience and technical success or serious AE rates. Additional analyses showed that institutional experience of ≤5 cases was an independent factor associated with prolonged procedure time, and that ascites was an independent predictor of serious AEs.

Several studies have reported the outcomes of EUS‐BD in low‐volume or inexperienced centers [12, 17, 18]. Koga et al. conducted a multicenter evaluation of EUS‐BD across 22 inexperienced centers, including 255 patients. To our knowledge, this is the largest study to date that specifically focuses on the implementation phase of EUS‐BD [12]. The cohort included EUS‐HGS and various EUS‐BD‐related procedures, such as EUS‐CDS, EUS‐guided hepaticoduodenostomy, EUS‐guided rendezvous technique, and EUS‐guided gallbladder drainage. Although their study demonstrated favorable clinical outcomes, the learning curve could not be evaluated owing to the heterogeneity of procedures and variations in case volumes across institutions.

Our study was limited to four institutions, each contributing 20 consecutive cases of EUS‐HGS, allowing for a focused assessment of the learning curve during the implementation phase—a notable strength of the present study. Analysis of the learning curves revealed that, although procedure time was significantly reduced with increasing institutional experience, no significant changes were observed in technical success or the incidence of serious AEs. Procedure time is often considered a surrogate marker of procedural proficiency, and the observed improvement can be interpreted as a positive indicator of technical maturation [19, 20, 21]. Additional analyses also showed that institutional experience of ≤5 cases was an independent factor associated with prolonged procedure time.

However, the lack of reduced technical failures and serious AEs with increased case volume suggests additional factors beyond procedural experience. Although no significant risk factors for technical failure were identified owing to the limited number of events, the presence of ascites was identified as an independent predictor of serious AEs. Patients with ascites often have a compromised general condition accompanied by hypoalbuminemia and sarcopenia, which may predispose them to a higher risk of serious AEs [22]. Moreover, ascites may exert a local negative effect on fistula formation and stabilization [23, 24]. In this study, no patient had massive ascites along the puncture tract, and ascites was not identified as a risk factor for technical failure. This suggests that EUS‐HGS may still be considered a therapeutic option if ascites is present, given that it does not occur along the puncture tract and the procedure is deemed tolerable and clinically beneficial. Nevertheless, as our results did indicate that ascites may increase the risk for serious AEs, the importance of careful preprocedural assessment must be emphasized.

Our clinical outcomes were equivalent to those of a systematic review and are favorable, even when considering differences between institutions and experience. These outcomes may be partially attributed to the substantial knowledge and experience of the participating endoscopists in ERCP and EUS‐related procedures. Sagami et al. reported that procedural outcomes may be influenced by the general experience with EUS‐related procedures [13]. Therefore, with substantial knowledge and experience in pancreatobiliary endoscopy, the implementation of EUS‐HGS may be feasible even in inexperienced centers. However, a serious AE rate of 11% cannot be overlooked. In our cohort, some serious AEs appeared unlikely to be directly attributable to the EUS‐HGS procedure itself, which may have contributed to the relatively high rate observed. Nonetheless, given that EUS‐HGS is an inherently high‐risk and technically demanding intervention, endoscopists must exercise particular caution when performing the procedure. This consideration is especially important as EUS‐HGS is increasingly implemented in clinical practice.

This study has some limitations. First, as the study was conducted in a specific region of Japan with only four participating centers, the outcomes may not be fully generalizable to global EUS‐HGS practices. Second, this study assessed the initial outcomes of EUS‐HGS during its implementation phase; therefore, long‐term outcomes, including recurrent biliary obstruction and the long‐term learning curve, were not assessed. Third, in the present study, procedure time decreased with increasing institutional experience. However, procedure time is influenced by multiple factors, including advancements in dedicated devices and changes in case selection. In this study, we used institutional experience because it reflects not only the accumulation of operator expertise but also the maturation of procedural proficiency and the growing familiarity of EUS‐HGS among assisting personnel and nursing staff. Nevertheless, because these factors cannot be fully controlled in a retrospective multicenter study, residual bias cannot be eliminated. Fourth, in our cohort, no cases demonstrated ascites along the puncture route; therefore, the safety of EUS‐HGS in this setting could not be directly evaluated. When ascites is present along the puncture route, EUS‐HGS may not only be associated with an increased risk of serious AEs but may also become technically more challenging. Fifth, owing to the insufficient number of events, the gauge of the puncture needle was not included in the analysis of technical failures or serious AEs. A 22‐gauge needle may facilitate puncture and be a viable option in EUS‐HGS [25, 26]. Finally, EUS‐HGS combined with antegrade stenting (EUS‐HGAS) was analyzed with EUS‐HGS. EUS‐HGAS is an effective treatment option as it can prolong the time to recurrent biliary obstruction [27, 28]. Because EUS‐HGS and EUS‐HGAS may have distinct risk profiles, separate analyses may be required to appropriately assess each procedure.

## 5 Conclusion

In this multicenter study, we demonstrated that EUS‐HGS can be feasibly implemented with caution in inexperienced centers, particularly when performed by experienced pancreaticobiliary endoscopists. Although procedure time decreased with increasing institutional experience, no improvement was observed in technical success or serious AE rates. Additionally, the presence of ascites was identified as an independent predictor of serious AEs.

## Author Contributions

**Junichi Kaneko** contributed to the study conception and design, video content creation, and data acquisition, analysis, and interpretation. **Tatsunori Satoh**, **Yosuke Kobayashi**, **Azumi Suzuki**, and **Shinya Kawaguchi** contributed to data acquisition and were involved in the final approval of the published version of the manuscript.

## Funding

The authors rececived no specific funding for this work.

## Ethics Statement

The study was approved by the Institutional Review Board of Iwata City Hospital (approval no of the Institutional Review Board: 2024–934).

## Consent

Consent for the use of the patient information was obtained through an opt‐out system.

## Conflicts of Interest

The authors declare no conflicts of interest.

## Clinical Trial Registration

Not applicable.

## Supporting information




**Figure S1**: Institutional learning curve of EUS‐HGS across five chronological quintiles: graph showing the relationship between technical success (A), procedure time (B), or adverse events (C) and institutional experience using five chronological quintiles (cases 1–4, 5–8, 9–12, 13–16, and 17–20). In (C), the overall events are indicated in black, while serious adverse events are indicated in gray.

**Table S1**: Details of patients who experienced technical failure or serious AEs.

## Data Availability

The datasets generated and/or analyzed during the current study are not publicly available due to restrictions related to participant confidentiality and privacy protection but are available from the corresponding author on reasonable request.
