# Supplementary figures and images for: Clinical Outcomes and Learning Curve of Endoscopic Ultrasound‐Guided Hepaticogastrostomy During the Implementation Phase in Inexperienced Centers: A Multicenter Retrospective Study
Source: DEN Open. 2026 Jan 31;6(1):e70291. doi: 10.1002/deo2.70291 (PMC12859524; doi:10.1002/deo2.70291)

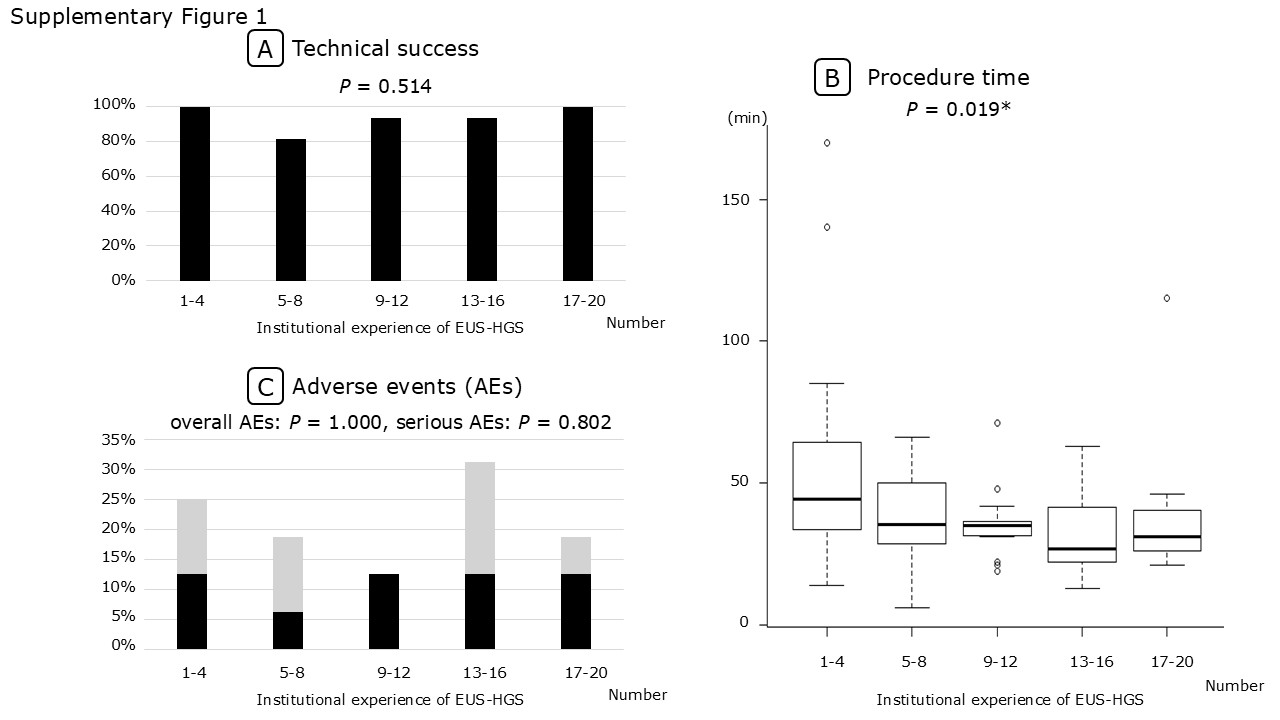

Supplement: Supplementary file 1 — Figure S1: Institutional learning curve of EUS‐HGS across five chronological quintiles: graph showing the relationship between technical success (A), procedure time (B), or adverse events (C) and institutional experience using five chronological quintiles (cases 1–4, 5–8, 9–12, 13–16, and 17–20). In (C), the overall events are indicated in black, while serious adverse events are indicated in gray. [file DEO2-6-e70291-s001.JPG]
