# Supplementary material for: Clinical Outcomes and Learning Curve of Endoscopic Ultrasound‐Guided Hepaticogastrostomy During the Implementation Phase in Inexperienced Centers: A Multicenter Retrospective Study
Source: DEN Open. 2026 Jan 31;6(1):e70291. doi: 10.1002/deo2.70291 (PMC12859524; doi:10.1002/deo2.70291)
Supplement: Supplementary file 2 — Table S1: Details of patients who experienced technical failure or serious AEs. [file DEO2-6-e70291-s002.docx]

| **Supplementary Table 1.** Details of patients who experienced technical failure or serious AEs | | |
| --- | --- | --- |
| Case No | Technical failure and/or serious AE | Details |
| 1 | Technical failure | Puncture was attempted with a 22-gauge needle, but cholangiography could not be obtained, resulting in EUS-HGS failure. PTBD was performed the following day, which resulted in clinical improvement. |
| 2 | Technical failure | Puncture and cholangiography were successful, but the guidewire could not be advanced, resulting in EUS-HGS failure. Because the patient had no jaundice, no additional biliary intervention was performed. |
| 3 | Technical failure | Puncture and guidewire placement were successful, but passage of the contrast catheter was unsuccessful, resulting in EUS-HGS failure. Because the patient had no jaundice, no additional biliary intervention was performed. |
| 4 | Technical failure and serious AE | Puncture and cholangiography were successful, but the guidewire did not advance into the bile duct, resulting in EUS-HGS failure. The patient developed hematemesis on postoperative day 2 and underwent emergency endoscopy with clip hemostasis and blood transfusion. The patient did not undergo additional biliary drainage due to poor overall condition. |
| 5 | Technical failure and serious AE | Puncture, guidewire placement, and catheter insertion were successful, but the procedure was aborted due to sudden respiratory arrest. Respiration resumed after scope withdrawal. Post-procedure, the patient developed aspiration pneumonia requiring antibiotic therapy, but no bile leakage was observed. The patient did not undergo additional biliary drainage due to poor overall condition. |
| 6 | Serious AE | Stent migration into the stomach was noted during stent indwelling, and biliary peritonitis occurred the following day. An additional metal stent was deployed through the HGS tract, resulting in clinical improvement. |
| 7 | Serious AE | During the procedure, the patient developed biliary bleeding, which required unplanned metal stent placement and a prolonged hospital stay for observation, resulting in clinical improvement. |
| 8 | Serious AE | During the procedure, the patient developed bleeding, and a subcapsular hepatic hematoma was identified on computed tomography the following day. Blood transfusion was required, but no intravascular intervention was needed. |
| 9 | Serious AE | On postoperative day 4, the patient developed cholangitis due to inadequate biliary drainage through the HGS tract and was treated with antibiotics and PTBD, which resulted in clinical improvement. |
| 10 | Serious AE | On postoperative day 4, the patient developed acute cholecystitis and underwent PTBD, which resulted in clinical improvement. |
| 11 | Serious AE | On postoperative day 4, the patient developed peritonitis with moderate ascites, resulting in a prolonged hospital stay for antibiotic treatment without additional intervention. |
| 12 | Serious AE | On postoperative day 13, the patient developed cholangitis and improved after additional biliary drainage via ERCP. |
| Abbreviations: AE, adverse event; EUS-HGS, endoscopic ultrasound-guided hepaticogastrostomy; ERCP, endoscopic retrograde cholangiopancreatography; PTBD, percutaneous transhepatic biliary drainage.  AE severity was graded according to the American Society for Gastrointestinal Endoscopy (ASGE) lexicon guidelines. | | |
